# Prognostic Value of the Acute Physiology and Chronic Health Evaluation II (APACHE II) Score in Secondary Peritonitis: A Narrative Review

**DOI:** 10.7759/cureus.105546

**Published:** 2026-03-20

**Authors:** Shubham R Kotwal, Dhaval V Patel, Chiral L Bamaniya

**Affiliations:** 1 Department of General Surgery, GMERS Medical College, Himmatnagar, Himmatnagar, IND

**Keywords:** acute generalized peritonitis, apache-ii score, intensive care unit, mortality prediction, prognostic scoring system, secondary bacterial peritonitis, surgical sepsis

## Abstract

Secondary peritonitis is a major surgical emergency worldwide and is associated with high morbidity and mortality, particularly in resource-limited settings. It most commonly results from perforation of a hollow viscus, causing contamination of the peritoneal cavity and triggering a severe systemic inflammatory response that may progress to sepsis and multi-organ dysfunction. Early identification of high-risk patients and objective assessment of disease severity are essential for guiding clinical decision-making, optimizing resource utilization, and improving outcomes. However, the lack of a precise and universally accepted classification system for acute generalized peritonitis limits accurate prognostic evaluation, and crude morbidity and mortality data alone are often inadequate for meaningful clinical audit.

Prognostic scoring systems, therefore, play a crucial role in the management of critically ill surgical patients. Among these, the Acute Physiology and Chronic Health Evaluation II (APACHE II) score remains one of the most widely used and extensively validated tools. It provides an objective measure of disease severity based on acute physiological variables, age, and chronic health status, using data obtained within the first 24 hours of intensive care unit (ICU) admission. Its simplicity, reproducibility, and applicability across diverse ICU populations have sustained its global use despite the availability of newer models.

This narrative review evaluates the role of the APACHE II in patients with secondary peritonitis based on previously published literature. Available studies generally demonstrate a positive association between higher APACHE II scores and adverse outcomes, including increased mortality, prolonged ICU and hospital stay, higher postoperative complication rates, and a greater need for re-intervention. However, reported prognostic cut-off values vary across different populations, study designs, and clinical settings, particularly between ICU-based cohorts and broader surgical populations.

Comparative studies suggest that while disease-specific scores such as the Mannheim Peritonitis Index may offer advantages in certain clinical contexts, APACHE II provides a broader physiological assessment. It is important to note that the score was originally developed and validated primarily in ICU settings where complete physiological data within the first 24 hours are available. When interpreted alongside clinical judgment, APACHE II remains a useful tool for risk stratification and prognostic assessment in patients with secondary peritonitis.

## Introduction and background

Peritonitis

Peritonitis refers to inflammation of the serous membrane lining the abdominal cavity and visceral organs. Based on the origin of infection, it is classified into primary, secondary, and tertiary types. Primary peritonitis occurs without visceral perforation. Secondary peritonitis results from perforation of a hollow viscus and represents the most common form worldwide. Tertiary peritonitis refers to persistent or recurrent infection following inadequate control of secondary peritonitis [1].

It is important to highlight that, unlike the findings commonly reported in Western literature, which indicate a higher prevalence of lower gastrointestinal tract perforations, cases observed in India and the subcontinent predominantly involve perforations of the upper gastrointestinal tract. Secondary peritonitis is primarily characterized by acute generalized peritonitis, a critical condition that represents a significant surgical emergency on a global scale. This condition is frequently encountered in the realm of general surgical practice and is associated with substantial morbidity and mortality rates [1-6]. Despite advances in surgical and critical care management, outcomes are largely determined by the severity of systemic inflammatory response and organ dysfunction at presentation.

Prognostic Assessment in Secondary Peritonitis

Early and objective assessment of disease severity is crucial for identifying high-risk patients, guiding therapeutic decisions, and optimizing resource allocation in critically ill individuals [7-9]. Reliable prognostic tools are therefore essential for risk stratification and outcome prediction in this population. Accurate severity assessment is particularly important in secondary peritonitis, where clinical presentation may vary widely, and early deterioration is common. The objective evaluation of severity, therapeutic approach, and effectiveness of treatment of acute generalized peritonitis from perforation is hampered by the lack of precise classification in this environment. Crude morbidity and mortality data for the purpose of medical audit are often misleading. Early prognostic evaluation is desirable to be able to select high-risk patients for more aggressive treatment, especially in severe peritonitis [10-12].

Scoring Systems in Peritonitis

Numerous scoring systems have proven effective in predicting outcomes for critically ill patients, thereby facilitating the optimal allocation of resources for enhanced efficacy [2]. The assessment of acute physiological conditions is frequently conducted utilizing a range of scoring systems, including the Surgical Infection Stratification System (SIS) and the Acute Physiology and Chronic Health Evaluation score (APACHE II). Furthermore, the Simplified Acute Physiology Score (SAPS) and the Sepsis Severity Score are often employed in clinical evaluations. In the specific context of peritonitis assessment, instruments such as the Mannheim Peritonitis Index (MPI) and the Altona II Peritonitis Index are regularly utilized [10,13,14].

APACHE II

The APACHE II score, developed by Knaus and colleagues in 1985, is widely regarded as one of the most extensively employed prognostic scoring systems for critically ill patients admitted to intensive care units (ICUs) [13]. The purpose of this instrument is to assess the severity of disease and to predict hospital mortality based on objective physiological data collected within the first 24 hours of ICU admission. The APACHE II score is derived from three primary components: the Acute Physiology Score (APS), which is calculated from 12 routinely measured physiological variables. These variables include body temperature, oxygenation, respiratory rate, heart rate, mean arterial pressure, arterial pH, serum sodium, potassium, creatinine, hematocrit, white blood cell count, and the Glasgow Coma Scale (GCS). Additionally, the scoring system incorporates age points to account for the increased risk of mortality associated with advancing age, as well as chronic health points that are assigned to patients exhibiting severe chronic organ insufficiency or who are in immunocompromised states [13]. The total score for the APACHE II system ranges from 0 to 71, with higher scores indicating greater severity of illness and an increased risk of mortality. The calculation of the APACHE II score involves the assignment of weighted points to each physiological variable, depending on the extent of deviation from normal values. The cumulative score is associated with predicted mortality rates, which are derived from regression equations that have been validated using extensive multicenter datasets [13,15]. The scoring system’s simplicity and reproducibility have contributed to its extensive global use across both surgical and non-surgical ICU populations [16,17]. The APACHE II score has been validated through extensive research and continues to be the most widely utilized prognostic system for evaluating critically ill patients. This is attributable to its straightforward application, objectivity, and established capability to predict patient outcomes effectively [3-5]. It is used for mortality prediction, risk stratification, ICU performance evaluation, and clinical research benchmarking [15-17]. Although newer models (e.g., SAPS II, Sequential Organ Failure Assessment (SOFA), APACHE IV) have emerged, APACHE II continues to be widely adopted owing to its strong validation record and clinical practicality [17,18].

While several disease-specific and general critical care scoring systems are available, their predictive accuracy and applicability vary across different clinical settings. Among these, the APACHE II scoring system has gained widespread acceptance due to its validated prognostic performance, simplicity, and applicability across diverse ICU populations. Therefore, this review specifically focuses on evaluating the role of APACHE II in secondary peritonitis.

Rationale: why the APACHE II score is relevant in peritonitis patients

In patients with secondary peritonitis, systemic inflammatory response and multi-organ dysfunction significantly influence outcomes. The APACHE II score incorporates acute physiological variables that reflect systemic derangements, making it particularly relevant in this context. Unlike purely anatomical or disease-specific indices, APACHE II captures the overall physiological impact of intra-abdominal sepsis. This characteristic enhances its utility in mortality prediction, ICU triage, and risk stratification among patients with secondary peritonitis [15,19,20].

This article is a narrative review aimed at summarizing and critically analyzing the existing literature on the prognostic utility of the APACHE II score in secondary peritonitis. Relevant studies were identified through searches of major medical databases, and key publications were selected based on their clinical relevance and contribution to the topic.

Objective of the current review

This study aims to critically evaluate and summarize the current literature regarding the application of the APACHE II scoring system in patients diagnosed with secondary peritonitis. The objective is to assess its correlation with clinical outcomes, including morbidity, mortality, and the duration of hospital or ICU stay. This review intends to underscore the prognostic value, accuracy, and clinical utility of the APACHE II score in risk stratification and outcome prediction for patients experiencing secondary peritonitis.

## Review

Methodology

Study Design

This study was conducted as a narrative review to critically evaluate and synthesize the existing literature regarding the prognostic utility of the APACHE II score in patients with secondary peritonitis. Given the significant methodological heterogeneity and variations in study design within the field, a narrative approach was selected to provide a comprehensive physiological assessment and qualitative summary rather than a meta-analysis.

Search Strategy

A comprehensive search was performed across major medical databases and electronic resources (including PubMed/MEDLINE and Google Scholar) to identify relevant studies. The search utilized a combination of the following keywords: "Acute eneralized peritonitis, APACHE-II score, Intensive Care Unit, Mortality prediction, Prognostic scoring system, Secondary bacterial peritonitis, Surgical sepsis."

Selection Criteria

The selection spanned foundational studies from the 1980s to contemporary research (1969-2025), including comparative analyses with other systems like the MPI and the SOFA. The review included studies that met the following criteria: patients diagnosed with secondary peritonitis, primarily resulting from hollow-viscus perforation (e.g., perforated appendicitis, peptic ulcers, or ischemic bowel); use of the APACHE II scoring system to evaluate disease severity within the first 24 hours of ICU admission; studies reporting on clinical outcomes such as hospital or ICU mortality, duration of stay, postoperative complications, and need for re-intervention; and literature from both developed and developing nations was included to account for differences in clinical burden and resource availability.

Data Synthesis and Analysis

Data were extracted and synthesized narratively, focusing on the correlation between APACHE II scores and adverse outcomes. The synthesis addressed specific clinical nuances, including threshold/cut-off values, the impact of timing on score calculation, and the role of subgroup analyses (e.g., elderly patients and comorbidities). Finally, the review incorporated future perspectives by examining the integration of APACHE II variables into machine learning (ML) and AI-enhanced prediction models.

Pathophysiology and clinical burden of secondary peritonitis

Etiology and Common Causes

Secondary peritonitis refers to inflammation of the peritoneal cavity due to contamination by gastrointestinal contents following a breach in the integrity of the gastrointestinal tract. It is most commonly caused by perforated appendicitis, peptic or duodenal ulcer perforation, traumatic bowel perforation, ischemic bowel, or anastomotic leakage following surgery [21,22]. The release of bacteria, digestive enzymes, and toxins results in a significant systemic inflammatory response, which can lead to sepsis, multi-organ dysfunction, and elevated mortality rates if not managed promptly [23].

Clinical Presentation and Challenges in Management

Patients with secondary peritonitis typically present with acute abdominal pain, rigidity, fever, tachycardia, hypotension, and signs of systemic sepsis. Diagnosis often requires a combination of clinical assessment, imaging (CT abdomen), and laboratory investigations. Despite advances in antibiotics, imaging, and critical care, management remains challenging due to delays in diagnosis, variable clinical presentation, antimicrobial resistance, and the need for urgent surgical intervention [24,25]. Early recognition and timely resuscitation remain key determinants of survival.

Mortality and Morbidity Trends in Developing vs. Developed Countries

Mortality rates from secondary peritonitis vary significantly worldwide. In developed countries, advances in critical care and early intervention have reduced mortality to approximately 10-20%, whereas in developing nations, rates remain as high as 20-60% due to late presentation, resource limitations, and inadequate perioperative care [11,26]. These disparities highlight the urgent need for simple, validated prognostic indicators that can be applied universally.

The Necessity for Reliable Prognostic Tools

Given the heterogeneous presentations and varied outcomes associated with peritonitis, the development of reliable prognostic scoring systems is crucial for the early identification of high-risk patients and the formulation of effective management strategies. The APACHE II score provides an objective and quantitative assessment of physiological derangements and has been extensively validated in critically ill patients, including those diagnosed with peritonitis [27,28]. The application of this approach aids clinicians in the processes of risk stratification, resource allocation, and outcome prediction. These contributions ultimately enhance patient care and facilitate comparability in research [3,14,29].

APACHE II score: concept and utility

The APACHE scoring system was developed by Knaus et al. in 1981. This system functions as an objective instrument for evaluating disease severity in critically ill patients and for estimating prognosis based on the extent of physiological abnormalities observed [30]. The original APACHE I model underwent refinement to develop APACHE II in 1985. This iteration incorporated a reduced number of physiological variables that are more clinically relevant, thereby enhancing both simplicity and reproducibility in its application [13]. The subsequent iterations, APACHE III (1991) and APACHE IV (2006), were developed to improve predictive accuracy by utilizing more extensive datasets and logistic regression modeling, particularly within diverse populations in ICUs [31,32].

The APACHE II score is derived from three major components: (1) APS: based on 12 routinely measured physiological variables - temperature, oxygenation (PaO_2_ or A-aDO_2_), respiratory rate, heart rate, white blood cell count, mean arterial pressure, serum sodium, potassium, creatinine, hematocrit, arterial pH, and GCS; (2) Age points: additional points assigned according to age category, reflecting increased mortality risk with advancing age; (3) Chronic health evaluation: points added for patients with severe chronic organ insufficiency or immunocompromised states (e.g., cirrhosis, heart failure, renal failure, malignancy) [13]. The total score ranges from 0 to 71, with higher scores indicating an increased severity of illness and an elevated projected mortality rate.

In the context of surgical sepsis and intra-abdominal infections, including secondary peritonitis, the APACHE II score has been established as a reliable instrument for risk stratification and predicting clinical outcomes [12,17]. Numerous studies have established a significant positive correlation between elevated APACHE II scores and adverse clinical outcomes. These outcomes encompass an increase in postoperative mortality rates, prolonged durations of stay in the ICU, and a heightened incidence of multiple organ dysfunction [12,33,34]. This process aids in identifying patients at high risk who may necessitate prompt surgical intervention, rigorous monitoring, or early admission to the ICU.

The advantages of the APACHE II scoring system include its extensive validation across diverse ICU populations, its straightforward calculation process, and its objective approach to evaluating the severity of physiological conditions [13,17,32]. The system has emerged as a benchmark tool for clinical research, ICU audits, and quality comparisons across various institutions. Nevertheless, it is important to acknowledge the limitations inherent in this system. It relies on data collected within the initial 24 hours of patient admission to the ICU, which may lead to an underestimation of dynamic changes in clinical status. Additionally, the requirement for comprehensive physiological data presents challenges, particularly in resource-limited settings [15,32]. Furthermore, the model’s calibration may vary across populations and disease profiles, warranting periodic local validation [15,31].

Correlation of APACHE II with outcomes in secondary peritonitis

Review of Studies Showing Correlation With Mortality, ICU Stay, Complications, and Re-operation Rates

Numerous observational studies have established a positive correlation between elevated APACHE II scores upon admission and adverse outcomes associated with secondary peritonitis. These outcomes include increased mortality rates, extended lengths of stay in both the ICU and hospital, heightened rates of postoperative complications, and an increased necessity for reoperation. Research focusing on hollow-viscus perforation and perforative peritonitis has indicated that the mean APACHE II scores among non-survivors are significantly higher than those observed in survivors. Moreover, specific score categories, particularly those ranging from 11 to 20 and exceeding 20, are associated with a markedly increased risk of mortality. Both serial and admission assessments of APACHE II scores have proven to be reliable indicators for predicting prolonged ICU stays and subsequent postoperative organ dysfunction [35,36].

Subgroup Analyses (Elderly, Comorbidity, Type of Perforation)

Subgroup analyses in multiple cohorts demonstrate that APACHE II retains prognostic relevance across age and comorbidity strata but that its absolute predictive performance and thresholds vary. Elderly patients and those with significant chronic comorbidities (e.g., cardiac, hepatic, renal failure) show higher baseline APACHE II scores and correspondingly worse outcomes for a given score compared with younger, otherwise healthy patients. Some studies report diagnostic performance differences by type of perforation (e.g., perforated peptic ulcer vs. ischemic or malignant perforation), with higher scores and worse outcomes in ischemic or delayed-presentation perforations. These findings support using APACHE II together with clinical context and disease-specific characteristics [37].

Threshold/Cut-Off Values Reported in the Literature

Published series report several pragmatic cutoffs linked to increased risk: many studies identify APACHE II values in the low-teens to mid-teens as the point where mortality risk begins to rise appreciably, with substantially higher mortality reported for scores >15-20. Some reports describe near-zero mortality at very low scores (<5-10) and steeply increasing observed mortality for scores >15, with several small cohorts reporting extremely high mortality when APACHE II exceeds 20. However, recommended cutoffs vary between populations and study settings, so local validation is advisable before adopting a rigid threshold for clinical decisions [35,36].

Comparison With Other Scoring Systems (MPI, SOFA, SAPS II)

Disease-specific scores such as the MPI are often more parsimonious and, in some series, outperform general ICU scores for predicting mortality in peritonitis, whereas APACHE II provides a broader physiologic assessment and is useful for benchmarking across heterogeneous ICU populations. Comparative studies show both MPI and APACHE II have good predictive value; some reports favour APACHE II for discrimination in mixed surgical populations and for use in risk-adjusted research, while others find MPI superior for pure peritonitis cohorts. Advantages and limitations depend on available data, timing of measurement, and intended use (individual triage vs. group-level benchmarking). Combining disease-specific and physiology-based scores (or using serial APACHE II measurements) can improve prognostic accuracy [11,12].

Factors influencing predictive accuracy

The timing of scoring plays a critical role in the predictive accuracy of the APACHE II scoring system. APACHE II is derived from the most severe physiological measurements recorded during the initial 24 hours following admission to the ICU. Consequently, discrepancies between values recorded at admission and those obtained at later time points, such as day 3 or upon discharge, may yield varying prognostic insights. Numerous studies have demonstrated that serial or subsequent assessments using the APACHE II score generally exhibit greater accuracy in predicting mortality than a singular score taken at admission [38,39].

Resource-Limited Settings vs. High-Resource ICUs

Prognostic models developed within high-resource environments, such as the APACHE cohorts, may exhibit diminished calibration and variable discrimination when applied to low- and middle-income countries (LMICs). This discrepancy can be attributed to differences in case mix, available interventions, and the completeness of data. External validation studies underscore the necessity for local recalibration or cautious interpretation of predicted mortality rates in resource-constrained settings [40].

Differences in Predictive Value Between Developed and Developing Nations

Comparative series and multicentre validations report that APACHE II generally retains good discrimination (area under the receiver operating characteristic (ROC), often 0.8-0.86) in high-income settings but shows wider variation in observed versus predicted mortality in other regions, reflecting delayed presentation, differing etiologies, and limited ICU capacity, so reported predictive accuracy and optimal cutoffs may differ by region [41].

Effect of Surgical Intervention Strategies (Damage-Control vs. Definitive Surgery)

Surgical strategy modifies physiology and subsequent scoring: Damage-control approaches are increasingly used for patients with severe sepsis/physiologic derangement and may improve short-term survival but also change postoperative physiology that underlies APACHE II calculations. Studies of non-trauma emergency laparotomy/peritonitis report that APACHE II (calculated pre- or immediately post-op) can help identify candidates for damage-control surgery, yet timing of measurement relative to the operation influences predictive accuracy [42,43].

Practical Implications

Use APACHE II as a dynamic, contextual tool; prefer serial measurements (or consider day 3/discharge scores when available), validate/calibrate the model locally in resource-limited settings, and interpret scores alongside surgical strategy, timing of source control, and clinical judgement rather than as a sole arbiter of care [39,44,45].

Clinical implications

The APACHE II scoring system serves as an invaluable clinical tool that assists surgeons and intensivists in multiple facets of patient care, ranging from early risk assessment to outcome evaluation and quality improvement.

Risk Stratification

By quantifying the degree of physiological derangement, the APACHE II score enables clinicians to objectively stratify patients according to disease severity at presentation [13,17]. In patients with secondary peritonitis, higher APACHE II scores have consistently correlated with increased postoperative complications, longer ICU stay, and higher mortality [3,12]. This helps identify high-risk patients who require early and aggressive resuscitation, close monitoring, and multidisciplinary management.

Decision-Making (ICU Admission and Aggressive Interventions)

The APACHE II score functions as an essential instrument for clinical triage and resource allocation, particularly within emergency surgical contexts where the capacity of the ICU is limited. Patients exhibiting elevated APACHE II scores are more likely to benefit significantly from increased monitoring, advanced hemodynamic support, and timely interventions aimed at source control [46]. Studies have shown that using physiologic scoring systems like APACHE II during ICU admission helps prioritize high-risk patients and guide escalation of care strategies, including aggressive surgical or damage-control approaches in severe peritonitis or septic shock [44,46,47].

Prognostic Counseling of the Family

Because the score correlates closely with hospital mortality, it provides clinicians with an objective framework for discussing prognosis with patients’ families [17,46]. This approach fosters transparent communication regarding anticipated outcomes, potential complications, and the necessity for extensive interventions, particularly in critically ill or elderly patients who present with multiple comorbidities.

Audit and Quality Assessment in Surgical Units

Beyond individual patient management, APACHE II serves as an audit and benchmarking tool to evaluate ICU and surgical performance across institutions. It allows comparison of observed versus predicted mortality (standardized mortality ratio) and has been used globally to monitor quality of care, evaluate outcomes over time, and assess the impact of new interventions [13,15]. Its simplicity and reproducibility make it one of the most widely accepted systems for quality improvement and performance benchmarking in surgical and critical care settings [15,48].

Limitations of current evidence

Although a considerable amount of literature substantiates the prognostic significance of the APACHE II score in cases of secondary peritonitis and other intra-abdominal infections, the existing evidence exhibits several notable limitations that affect both the interpretation and generalizability of the findings.

Heterogeneity in Studies

Published studies vary widely in study design, inclusion criteria, and patient populations, ranging from localized appendicular peritonitis to generalized fecal peritonitis and tertiary sepsis, resulting in significant methodological heterogeneity [3,11,12]. Differences in case mix, surgical strategy, antimicrobial protocols, and critical care resources contribute to variability in reported mortality and the discriminative ability of APACHE II, limiting direct comparison and meta-analytic synthesis [3,12].

Sample Size

Many studies evaluating APACHE II in peritonitis are single-center, retrospective analyses with relatively small cohorts (often <150 patients), which reduces statistical power and external validity [3,27,36]. The predominance of small observational series also increases susceptibility to selection bias and underrepresentation of elderly or comorbid subgroups.

Lack of Standardization in the Timing of Score Calculation

There is no universal consensus on when APACHE II should be calculated in surgical sepsis, whether preoperatively, immediately postoperatively, or after ICU admission, which affects predictive accuracy and comparability across studies [15,38,44]. Several authors have demonstrated significant variability in mortality prediction depending on the timing of scoring, underscoring the need for standardization in methodology.

Need for Multicentric, Large Prospective Studies

Few large, multicentric prospective trials have specifically assessed APACHE II performance in secondary peritonitis. Most validations originate from general ICU datasets rather than disease-specific cohorts. Future multicenter, prospective studies incorporating standardized timing, uniform definitions, and integration with disease-specific indices (e.g., MPI) are needed to establish robust cut-offs and improve calibration across diverse healthcare settings [3,15,40].

Future perspectives

While the APACHE II score remains one of the most extensively validated and widely applied prognostic tools in critical care, evolving clinical practices, changing patient demographics, and advances in computational modeling necessitate continuous refinement of prognostic systems for conditions such as secondary peritonitis.

Integration With Newer Scoring Systems

Recent studies emphasize the significance of employing the APACHE II scoring system in conjunction with various assessment tools, such as the SOFA, SAPS II, and the MPI. This integrated approach seeks to enhance prognostic accuracy and to effectively monitor the progression of physiological changes over time [3,17,49]. Combining dynamic organ dysfunction scores like SOFA with baseline physiologic severity measures such as APACHE II provides better mortality prediction in sepsis and intra-abdominal infections [49,50].

Dynamic and ML-Based Prediction Models

Contemporary research has explored ML and artificial intelligence (AI) models that integrate APACHE II variables with real-time electronic health record (EHR) data to enhance prediction accuracy [51,52]. ML algorithms, such as random forests and neural networks, have exhibited enhanced performance in comparison to traditional models when forecasting mortality rates, length of stay in the ICU, and postoperative complications. Additionally, these algorithms can retain their interpretability through the application of explainable AI frameworks [51,53]. Future systems may use continuous physiological monitoring and biochemical data to generate dynamic, patient-specific risk estimates that adapt with clinical changes.

Regional Calibration and Validation

Given the variability in healthcare resources, case mix, and disease burden, regional recalibration of APACHE II is essential. Studies from LMICs indicate reduced calibration and overprediction of mortality compared to high-resource ICUs [17,40]. Large, multicentric prospective studies that include diverse populations from both developed and developing nations are needed to establish locally validated cut-offs and predictive models tailored to specific healthcare environments [17,28].

Integration Into Digital Clinical Decision Support Systems (CDSS)

The incorporation of APACHE II and related models into digital ICU dashboards and EHR-based CDSS platforms can facilitate automated score computation and assist in real-time risk stratification, triage, and resource allocation [51,54]. Future clinical pathways may employ hybrid systems where AI-enhanced APACHE II models provide continuous, explainable, and actionable prognostic support to clinicians managing peritonitis and sepsis.

## Conclusions

The future direction of prognostic modeling in secondary peritonitis should aim at standardizing scoring protocols, enhancing real-time predictive capability, and promoting global equity in the applicability of critical care scoring systems. Integration of APACHE II with organ dysfunction scores and ML models holds promise for precision prognostication and personalized care in surgical sepsis.
